# Hexagonal-Grid-Layout Image Segmentation Using Shock Filters: Computational Complexity Case Study for Microarray Image Analysis Related to Machine Learning Approaches

**DOI:** 10.3390/s23052582

**Published:** 2023-02-26

**Authors:** Aurel Baloi, Carmen Costea, Robert Gutt, Ovidiu Balacescu, Flaviu Turcu, Bogdan Belean

**Affiliations:** 1 Research Center for Integrated Analysis and Territorial Management, University of Bucharest, 4-12 Regina Elisabeta, 030018 Bucharest, Romania; 2 Faculty of Administration and Business, University of Bucharest, 030018 Bucharest, Romania; 3 Department of Mathematics, Faculty of Automation and Computer Science, Technical University of Cluj-Napoca, 400114 Cluj-Napoca, Romania; 4 Center of Advanced Research and Technologies for Alternative Energies, National Institute for Research and Development of Isotopic and Molecular Technologies, 400293 Cluj-Napoca, Romania; 5 Department of Genetics, Genomics and Experimental Pathology, The Oncology Institute, Prof. Dr. Ion Chiricuta, 400015 Cluj-Napoca, Romania; 6 Faculty of Physics, Babes-Bolyai University, 400084 Cluj-Napoca, Romania

**Keywords:** hexagonal grids, shock-filter, machine learning, image segmentation, computational complexity, gene expression, microarray

## Abstract

Hexagonal grid layouts are advantageous in microarray technology; however, hexagonal grids appear in many fields, especially given the rise of new nanostructures and metamaterials, leading to the need for image analysis on such structures. This work proposes a shock-filter-based approach driven by mathematical morphology for the segmentation of image objects disposed in a hexagonal grid. The original image is decomposed into a pair of rectangular grids, such that their superposition generates the initial image. Within each rectangular grid, the shock-filters are once again used to confine the foreground information for each image object into an area of interest. The proposed methodology was successfully applied for microarray spot segmentation, whereas its character of generality is underlined by the segmentation results obtained for two other types of hexagonal grid layouts. Considering the segmentation accuracy through specific quality measures for microarray images, such as the mean absolute error and the coefficient of variation, high correlations of our computed spot intensity features with the annotated reference values were found, indicating the reliability of the proposed approach. Moreover, taking into account that the shock-filter PDE formalism is targeting the one-dimensional luminance profile function, the computational complexity to determine the grid is minimized. The order of growth for the computational complexity of our approach is at least one order of magnitude lower when compared with state-of-the-art microarray segmentation approaches, ranging from classical to machine learning ones.

## 1. Introduction

In digital-image processing and computer vision, image segmentation represents the process of dividing an image into multiple segments, representing non-overlapping pixel areas with homogeneous features. The resulting image segments are meaningful for defining objects according to human visual perception within the image under analysis. In biomedical and material science applications, when digital images are used to characterize either multiple biological samples or material structural patterns, the image segments (objects) are often disposed using a grid layout. By the grid layout, one can understand a network of lines that cross each other to form a series of geometrical figures which confine all image objects according to their pattern. Hexagonal grid layouts are used when printing space needs to be efficiently managed. An eloquent example is the microarray technology, where the hexagonal grid is considered advantageous compared to the rectangular grid, since it allows more DNA specific probes to be printed onto the same surface [1]. Moreover, images illustrating the hexagonal grid layout of the material structure are registered in cases of different applications. In cell-cluster-array fabrication, self-assembled hexagonal superparamagnetic cone structures induce a local magnetic field gradient which inhibits the cancer cells’ migration [2]. In material science applications, benefits such as increased optical performance or material resistance are added by hexagonal grid structures. The performances of the pixelated CsI(Tl) scintillation screens in X-ray imaging are enhanced by using a hexagonal array structure for the micro-columns’ shapes [3]. Microlens arrays consisting of circular nanostepped pyramids disposed in hexagonal arrangements have shown efficient bidirectional light focusing and maximal numerical apertures [4]. Considering the above cases, imaging techniques such as grid alignment and registration can be employed to determine the locations of objects in images. After targeting the resulting locations, further analysis by means of image segmentation is performed in order to extract the features of the image objects. Much research effort has been devoted to the development of image segmentation methods, and a wide range of applications exist in the field of image analysis and understanding. In medical image analysis for example, segmentation plays an important role in tasks such as visualization, measurement and reconstruction of shapes and volumes [5,6,7]; medical diagnosing [8,9]; and even image guided-surgery [10]. Recent research has proposed a large variety of techniques for image segmentation, which can be mainly classified as region-based segmentation, feature-based clustering or machine learning ML-based segmentation. Clustering-based techniques divide the image pixels based on their intensities into homogenous clusters while ignoring the spatial information, which makes them sensitive to image artifacts [11]. Considering its efficiency among the clustering-based algorithms, fuzzy C-means (FCM) has been widely used for image segmentation [12]. Improved variants which make use of the spatial information have been proposed to overcome the aforementioned limitations [13]. Regarding the machine learning approaches for image segmentation, both supervised and unsupervised ones are available. Unsupervised learning has the advantage of automatic segmentation without any prior knowledge of the object features within the training dataset [14]. Computationally expensive tools such as support vector machines, and probabilistic models such as Markov-random fields or Gaussian mixtures [15,16], are nevertheless used. The supervised ML techniques for image segmentation are more accurate and reliable, mainly since the input data are labeled and well known. Despite their computational complexity, deep learning algorithms, decision trees and Bayesian networks are broadly used in applied research [17]. Thus, computer-aided medical diagnosis is carried out on the basis of deep learning algorithms [18,19,20,21]. Bayesian networks [22,23,24] successfully conduct the detection of different geometries related to objects of interest in medical images, such as coronary arteries and retinal vasculature. A decision tree classifier can be used to obtain an adaptive threshold for the optic disc segmentation [25]. The advantages of both decision trees and conditional random fields have also been exploited [26,27]. In order to overcome the disadvantages of the supervised training, implicit deep supervision is assured by the hyper-densely connected convolutional neural network (CNN) proposed for natural image classification tasks [28,29], whereas level-set segmentation leads to semi-supervised CNN segmentation [30]. Considering various imaging technologies, there are cases when image objects are disposed of using a specific grid layout within the same image. In these cases, prior to image segmentation, a grid alignment or image registration procedure is mandatory. Thus, we focus on the registration and segmentation of hexagonal-grid-layout images.

As referred to the aforementioned image processing tasks (i.e., grid alignment and segmentation), when taking into account the large variety of state-of-the-art segmentation approaches, the main challenge is to choose the appropriate image-processing methods for feature extractions while considering both accuracy and computational complexity. In this context, the main findings are presented as follows.

### 1.1. Main Findings

The present paper proposes a set of image-transformation methods based on shock-filters applied on hexagonal-grid-layout images, aiming for both grid alignment and segmentation of the image objects. The PDE formalism of the shock filter, together with mathematical morphology, is used to evolve image profiles in order to determine the grid layout, by identifying a pair of sub-images, each containing a rectangular grid. The superposition of the two sub-images generates the initial image. Within each rectangular-grid-layout image, another procedure of profile evolution based on the same shock-filter formalism is used to confine the foreground information for each image object into an area of interest. For accurate segmentation of non-homogenous or irregular image objects, pixel intensity refinement classifies pixels to foreground or background, to better fit the true shape of the object. The proposed image-processing workflow was successfully applied to hexagonal-grid-layout microarray images, the hexagonal array structure of pixelated scintilation screens and hexagonal nanodisk–nanohole structure arrays. For microarray images classified as having a hexagonal grid layout, in spite of their advantages and intensive use [31,32], relatively few image-processing methods have been proposed. In [1], a spot-indexing algorithm successfully located microarray spots for hexagonal grids with different spacing and rotation. Giannakeas et al. also proposed a growing concentric hexagon algorithm [33], which detects spots in microarray images with a hexagonal grid layout. As compared with existing approaches, the main benefits of the proposed work are underlined as follows:

- The image-processing workflow represents a general solution for both rectangular and hexagonal grid alignment, which has been successfully applied to both medical images and images of material structures.
- The shock-filter-based grid alignment also delivers segmentation information, and guided by an autocorrelation procedure, it estimates the locations of missing objects within the hexagonal grid layout.
- The computational complexity required to determine the grid layout is minimized, taking into account that the PDEs are targeting the one-dimensional luminance function profiles,
- The segmentation accuracy was evaluated by computing the means and standard deviations of distances between the annotated and detected centers and showed improved results compared with state-of-the-art research.

In order to underline the main findings, the paper is organized as follows. Firstly, in the introductory section, the shock filters in the context of image segmentation and grid alignment are shortly summarized. Section 2 describes the shock-filter-segmentation approach applied for hexagonal-grid-layout microarray images. The results are shown in Section 3, in terms of segmentation accuracy, and the same section underlines the results obtained using the proposed methodology for two other types of hexagonal-layout images. In addition, the computational complexity of our approach is evaluated in the context of existing classical and machine learning solutions for grid alignment. Finally, the Conclusions section summarizes the main results.

### 1.2. Shock-Filter Fundamentals

An important task in image processing is to separate image areas containing background from foreground information. A shock-filter-based approach involves a process of selectively applying erosion or dilation in a localized manner in order to create a “shock” between two image areas, one belonging to a maximum and the other to a minimum. By iterating this process according to time increments, the resultant image reveals discontinuities only at the edges of the initial image. Moreover, the image areas delineated by the underlined edges become uniform in terms of pixel intensity values, delivering image segmentation information. Commonly, image enhancement processes, such as the one described before, are modeled through a partial differential equation (PDE).

Taking account of the importance of total variations in TV principles which appear for shock calculations in fluid dynamics, Osher and Rudin [34] have applied these ideas to image processing. This was revealed to be a useful method to restore discontinuities in images, such as edges. Their method relies on total variation techniques subject to a certain nonlinear and time-dependent partial differential equation:

(1) $$\partial_{t}u=-\left|\nabla u\right|F\left(L\left(u\right)\right),$$

where L(u) is a second-order, nonlinear elliptic operator whose zero-crossings correspond to edges. The filtering process (the edge enhancement process) is represented by the evolution of the initial image data $u_{0}\left(x\right)$ into a steady-state solution $u_{\infty }\left(x\right)$ as t→∞, through u(x,t),t>0. The total variation of the solution,

(2) $$TV\left(u\right):=\int_{D}\left|\nabla u\right|dx,$$

at any given state, is preserved and satisfies a maximum principle.

The steady state solution is achieved relatively fast, making it a good candidate for microarray image segmentation. As mentioned in [34], it is an O(kN) method, where *N* is the number of points and *k* the number of time iterations. It was pointed out by [35] that the one-dimensional Equation of (1) with F(u):=sgn(u), i.e.,

(3) $$u_{t}=-sign\left(u_{xx}\right)\left|u_{x}\right|,$$

is based on the image-enhancement algorithm of Kramer and Bruckner [36], which was proved to converge after a finite number of iterations.

From a morphological perspective, such a filter aims to produce a flow field which is directed from the interior of a region towards its edges, where it develops shock, generating a piecewise constant solution with discontinuities only at the edges of the original image. However, TV preserving methods suffer from fluctuations due to noise, which also create shocks. Therefore, Alvarez and Mazorra [37] considered the operator $L\left(u\right)=u_{xx}$ in (1) to be the Gaussian-smoothed version $L\left(G*u\right)=G*u_{xx}$, which supplemented the evolution with a noise-eliminating mean-curvature process, for which they proved that the discrete scheme is well-posed and satisfies a maximum—minimum principle. Smoothed morphological operators (dilations, erosions) for shock filters were also employed in [38] to enhance contours through smoothed ruptures, while preserving homogeneous regions.

## 2. Shock-Filter-Based Approach for Microarray Image Segmentation

Genes represent DNA sequences which determine particular characteristics in living organism, as follows: the genetic information is transmitted from nucleus to cytoplasm by an intermediate molecule called mRNA, which is further on translated into functional gene products known as proteins. Genes’ expression levels are reflected in the amounts of respective mRNA present in each cell, providing information on the cell’s biochemical pathways and its functions. By measuring mRNA levels for fully sequenced genomes printed on a solid surface, microarray technology is known to be a valuable tool for determining genes’ functionality and expression levels in different conditions [39].

The workflow of a microarray experiment aiming at gene expression estimation starts with labeling mRNA samples with different fluorescent markers and hybridized onto the same solid surface. Depending on researchers’ needs, gene expression analysis is performed by a one-color or a two-color experiment [40]. After hybridization, laser scanning is performed using one or two light sources with different wavelengths, one for each marker. The fluorescence induced by each light source is captured, and a composite image is produced. The microarray image thus obtained represents a collection of microarray spots, each spot corresponding to a specific gene.

Current technologies allow accurate fluorescence quantification [41], considering different numbers of spots at different densities printed onto a microarray slide, offering a broad view that represents all known genes and their transcripts in the human genome. Two spot layouts can be distinguished: the rectangular grid layout and the hexagonal grid layout, corresponding to the single-density and double-density microarrays, respectively. Commonly, microarray manufacturers use single-density microarrays, where spots are disposed in a rectangular grid. Nevertheless, taking into account that no matter the grid format, sensitivity and performance are preserved, the hexagonal grid is considered advantageous compared to the rectangular grid, since it allows more probes to be printed onto the same surface. Later-stage image-processing techniques, including object registration and segmentation, are used to estimate gene expression. Logical coordinates are determined for each spot of the microarray image, and the segmentation classifies pixels either as foreground, representing the DNA spots, or as background. A great deal of research has been conducted for processing microarray images having a rectangular grid layout. Bariamis et al. [42] used a SVM approach for automatic grid alignment. That, and an approach consisting of optimal multilevel thresholding, followed by a refinement procedure and hill climbing [43,44], lead to accurate grid detection. For spot segmentation, adaptive pixel clustering [45,46], snake fisher models [47,48], 3D spot modeling [49], bio-inspired algorithms [50] and Markov random field modeling [51] were proposed by state-of-the-art research. Nevertheless, considering the reduced publications tackling the hexagonal-grid-layout images [1,33,52], as underlined in the main findings sub-section, we propose a general approach for hexagonal- and rectangular-grid-layout microarray images.

### 2.1. Materials and Methods

**Figure 1:**
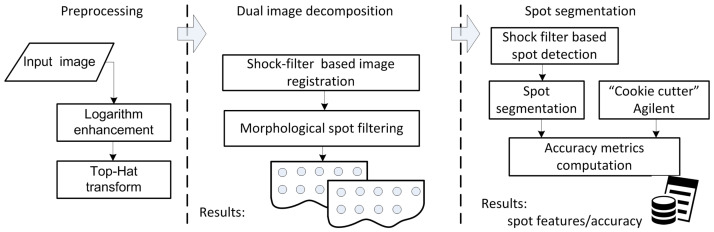
Image-processing workflow for hexagonal-grid-layout image registration and segmentation.

The microarray scanning process delivers 16x bit gray-scale images, in TIFF format, in which spot fluorescence levels are captured as intensities of the image pixels which fall within the microarray spot. To identify the position, intensity and background intensity values of each microarray spot, preprocessing techniques, image registration and image segmentation approaches are applied. The preprocessing methods aim at image enhancement based on logarithmic and top-hat image transforms to further improve the accuracy of spot detection. Further on, using the shock-filtered image profiles, each spot line is detected, and making use of a refinement procedure based on morphological filtering, the original image is decomposed into two sub-images, each of them containing a rectangular grid of spots. Next, the segmentation classifies pixels as belonging to the microarray spot or to the image background using the same PDE formalism specific to the shock filters, and the segmentation accuracy metrics are computed. The entire workflow can be depicted in Figure 1. The subsequent sub-sections detail the proposed image-processing techniques for automatic hexagonal-grid-layout microarray image analysis.

### 2.2. Preprocessing

**Figure 2:**
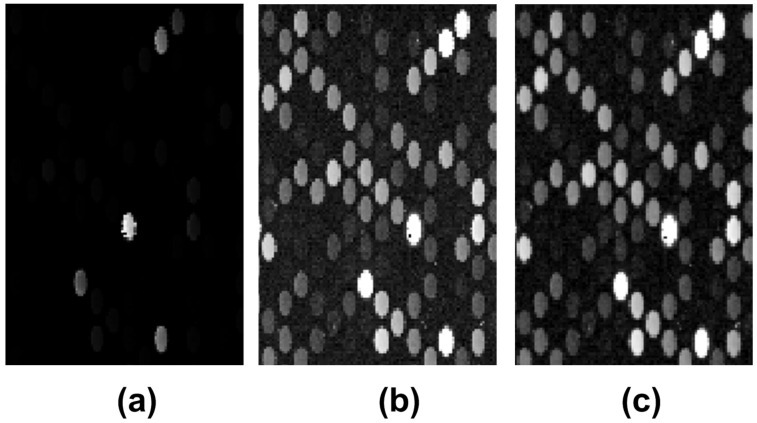
Image preprocessing techniques applied to the AT218398 microarray image: (**a**) original image, (**b**) logarithmically transformed and normalized image, (**c**) top-hat transformed image.

Weakly expressed spots and image rotation are common characteristics of the microarray images delivered by the scanning process. Thus, to enhance weekly expressed spots, a logarithm point-wise transform was applied on the image, followed by an intensity adjustment procedure so that the intensity histogram would fit the full dynamic range of the image (the dynamic range was from 1 to $2^{16}$). Moreover, a top hat transform was used to reduce the background influence on the microarray spots [53]. In case of misaligned input image, a rotation detection and correction algorithm (the Radon transform) was employed [54]. Figure 2 shows the results of the aforementioned preprocessing techniques for the US218398 microarray image.

### 2.3. Grid-Line Detection for Image Registration

Let $I_{P}=p_{i,j}$ be the preprocessed, M×N-pixel microarray image, with $p_{i,j}$ being the 16-bit intensity of the pixel found on row *i* and column *j* within the microarray image. The vertical image profile was computed as described by the equation

(4) $$V\left(i\right)=\frac{1}{N}\sum_{j=0}^{N-1}p_{i,j},\;i=0,\ldots ,M-1,$$

whereas the horizontal profile is described by

(5) $$H\left(j\right)=\frac{1}{M}\sum_{i=0}^{M-1}p_{i,j},\;j=0,\ldots ,N-1.$$

The vertical profile is evolved further on using the shock-filter partial differential Equation (3) given by

(6) $$u_{t}=-sign\left(u_{xx}\right)\left|u_{x}\right|,$$

where $u_{x}$ and $u_{xx}$ are the first- and the second-order derivatives of the image profile. The initial value of *u* at time t=0 is the image’s luminance function profile V(i).

**Figure 3:**
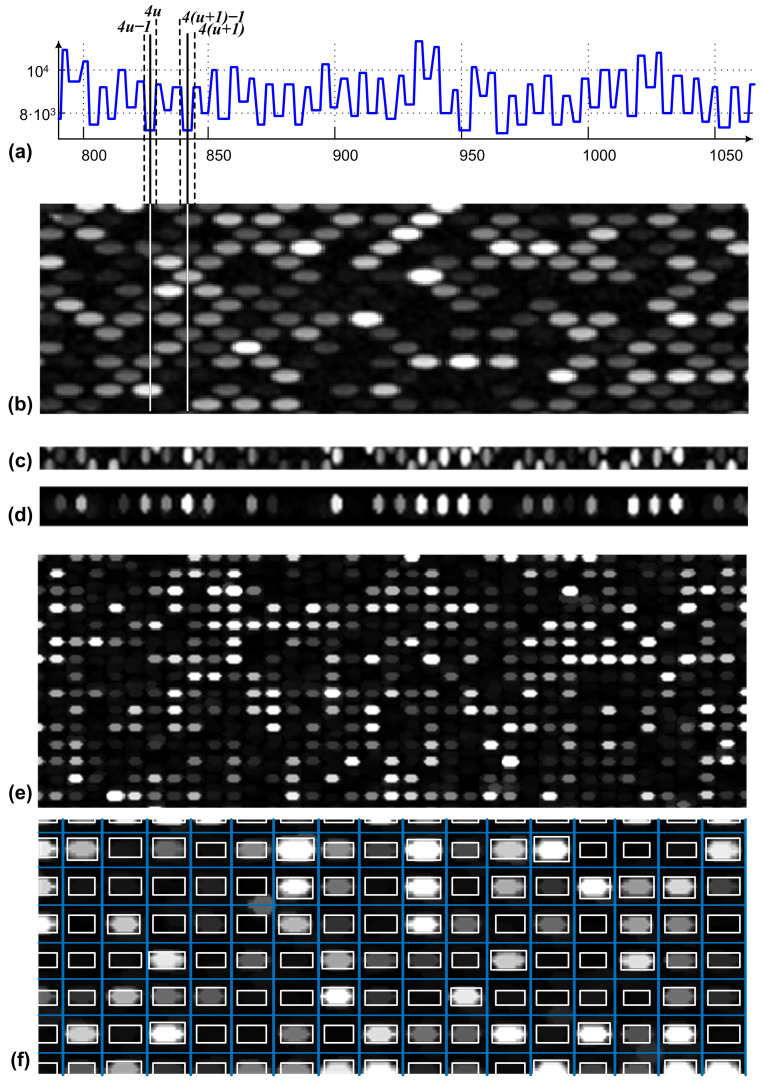
Hexagonal grid segmentation process: (**a**) horizontal profile of (**b**) the preprocessed microarray image, (**c**) selected lines based on separation lines, (**d**) morphological exclusion of neighboring spots, (**e**) final rectangular even spots $I_{ev}$ and (**f**) segmentation of the rectangular image.

Let the shock-filtered profile of the preprocessed microarray image vertical profile be denoted by SFP=V(i). The inflexions points are marked within the SFP, and their locations respect a specific pattern which reveals the borderlines for the separation of lines of spots. Figure 3 shows how the spots’ line separation is performed. The total number of lines of spots (see the line presented in Figure 3d), within the overall microarray image is considered to be *n*. The positions of all inflexion points detected along the profiles are stored in an uni-dimensional vector pos, and the resultant vector size is 2n. To define each line of spots, the positions of four inflexions points are considered. Thus, as shown in Figure 3a, the uneven lines of spots are defined as the positions 4u−1, 4u, 4(u+1)−1 and 4(u+1) within the pos vector. *u* ranges from 1 to n/2−1. In a similar manner, the positions 4u−3, 4u−2, 4u+1 and 4u+2 define the even lines of spots. The average position between 4u−1 and 4u and the average position between 4(u+1)−1 and 4(u+1) mark the positions of the horizontal separation’s lines for the uneven line of spots denoted by *u*. As presented in Figure 3b, the continuous lines over the $90^{0}$-rotated section of the original image are the separation lines for spot line *u*. Based on the aforementioned separation lines, all even and uneven lines of spots were detected. An example of such a line is presented in Figure 3c. It can be observed that the detected uneven lines of spots also include half of the spots within the neighboring even lines of spots. A similar situation describes the even lines of spots. In order to decompose the microarray image in two sub-images, one including the even lines of spots and the other one with the uneven line of spots, mathematical morphology is applied.

As denoted by Figure 3e, each microarray spot is defined by an elliptic shape characterized by the horizontal radius *a* and the vertical radius *b*, confined in a rectangular area. In the subsequent step, the average horizontal and vertical radii *a* and *b* considering all spots are estimated based on the autocorrelation applied on vertical and horizontal profiles of the image, described by Equations (4) and (5), respectively. A structuring element having an elliptical shape with *a* and *b* radii was defined. The upper and lower parts of each line of spots were padded with b/2 lines of pixels; each pixel has the lowest intensity value. The resulting image was morphologically opened with respect to the defined structural element. The outcome was similar to the lines of spots from Figure 3d, where the half spots from the neighboring lines were excluded. Further on, the original image was reconstructed once using the even lines of spots and once using the even lines of spots. Each resulted sub-image is characterized by a rectangular grid layout of its microarray spots (see Figure 3e). The sub-images containing the even lines of spots and uneven lines of spots are denoted by $I_{ev}$ and $I_{uev}$, respectively.

By applying the shock filter to the original vertical profile, the inflexion points at the positions 4u and 4(u+1)−1 within the vertical profile (see Figure 3a,d) can determine the locations on the vertical axes for all the spots from the column of spots denoted by *u*. Within the sub-image containing the column *u*, the horizontal profile *h*, as referred to in Figure 3d, is evolved using shock filters to determine the positions of spots on the *x* axis. The autocorrelation-based approach applied on the *h* image profile, described in [55], is used to estimate the positions of missing spots. Consequently, for each microarray spot position within the initial hexagonal grid, an area of interest, denoted by *S*, which confines the microarray spots, is determined according to Figure 3e. On each area *S*, image segmentation is applied next to determine the pixels which belong to the microarray spots and which belong to the spot’s local background. The aforementioned procedure is consistent with the “cookie cutter” approach used by the software platform Agilent Feature Extraction (FE) and detailed in [56].

### 2.4. Spot Segmentation

The shock filters deliver segmentation information by identifying simple geometric objects of rectangles for the entire set of microarray spots. For accurate segmentation of spots with spatial non-homogeneous intensity distribution and irregular shapes, a simple threshold procedure is introduced for the *S* area. As demonstrated in [55], pixels intensity refinement yields a rearrangement of pixels to the foreground and background that better fits the true shape of the spots.

## 3. Results and Discussions

Our study included a set of four microarray images used for one-color analysis of gene expression data performed using Agilent Technologies (G2505C scanner) on homo sapiens samples. The samples were printed on microarray glass slides formatted with four high-definition 44K, arrays and the images within the dataset have a hexagonal grid layout.

### 3.1. Microarray Image Registration and Segmentation Accuracy

We evaluate the results obtained using the proposed hexagonal grid alignment procedure compared with state-of-the-art results and with the results delivered by Agilent Feature Extraction Software (FE). Spot centers were annotated by FE for each microarray image from our dataset. The value $d_{i}$ representing the distance between an annotated spot’s center and the one determined using our proposed approach was computed for each spot (*i*) included in the image under analysis. The mass center’s locations ($m_{i}$) was determined for each spot $I_{i}$, and compared to the mass center’s location ($m_{i}^{FE}$) determined by FE software. The mean Euclidean distance $m_{E}$ between the two mass centers for the whole population of spots was used as a metric for the accuracy evaluation. The mean Euclidean distance $m_{E}$ was measured in pixels, and it is denoted by the equation

(7) $$m_{E}=\frac{1}{Ns}\sum_{i=1}^{Ns}\left|m_{i}-m_{i}^{FE}\right|,$$

Table 1 shows the average distance d=1.52 for the whole population of spots included in our dataset and underlines that the proposed approach delivered the lowest standard deviation for the distance *d* distribution over the whole population of spots compared with state-of-the-art hexagonal grid alignment approaches. The methodology’s accuracy given by the percentage of spots correctly positioned by the grid alignment procedure on the selected images was 100%.

Our proposed automatic image processing approach for hexagonal-grid-layout microarray images is evaluated in terms of accuracy and reproducibility, with regard to the whole population of spots within the microarray dataset. The mean spot intensity value $I_{i}$ was computed by subtracting the mean intensity value of the background pixels and the mean intensity value of pixels which fall within the microarray spots. The range of *i* is from 1 to $N_{S}$, where $N_{S}$ is the total number of spots within the microarray image. The results are compared with the ones delivered by the Agilent Feature Extraction software (FE) for the same set of images. Consequently, the accuracy estimation of our proposed segmentation method was performed independently on each microarray image from our dataset.

The regression ratio (R) represents an independent measure defined by the slope of the least-squares best-fit regression line of the fluorescence intensity values for each pixel against each other for a given microarray spot. The regression ratio indicates individual spot quality. Considering the regression pixels used to calculate *R* values, the coefficient of determination $R^{2}$ for the least-squares-regression fit of a microarray spot is defined as the square of the correlation coefficient and ranges in value between 0 and 1 [57]. For validating our approach, we correlated the coefficients of determination computed by our approach with the ones determined by FE for the entire population of microarray spots within each microarray image. Let $R^{2}$ be the coefficient of determination computed using the proposed approach and $R_{FE}^{2}$ be the coefficient of determination annotated by FE. The correlation coefficient, together with the mean difference between our results and the FE results, is described by:

(8) $$r=Pearson(R^{2},R_{FE}^{2}),$$

(9) $$agv_{diff}=\frac{1}{N_{s}}\sum_{i=1}^{N_{s}}\left|R_{i}-R_{FE}^{2}\right|,$$

The Pearson coefficient exceeded values of 0.98, and hence, indicated a high correlation of our data (intensities) with the reference values. Moreover, the reproducibility of the segmentation technique was quantified by means of mean absolute error MAE and coefficient of variation CV, as presented in Equations (10) and (11), according to [58,59], respectively. The lower the MAE and CV values are, the better the performance of the proposed method. r=4 replicates of the microarray experiment were used for evaluation. MAE indicates the spot sameness of the spot’s intensities, Equation (10), where $I_{j}$ is the mean spot intensity over the *j* experimental replicates and $\underline{I}$ is the overall mean, computed from the means of the spots within all the *r* replicates.

(10) $$MAE_{spot}=\frac{1}{r}\sum_{j=1}^{r}\left|I_{j}-\underline{I}\right|,$$

Spots intensity variations are expressed by the CV parameter denoted by Equation (11), based on the standard deviation σ of spot intensity with subtracted background and the mean spot intensity ν.

(11) $$CV_{spot}=\frac{\sigma }{\nu }.$$

The small CV values correspond to small variation among the pixel intensity values for given microarray spots, showing the reliability of the proposed grid alignment procedure together with the spot segmentation approach. As referred to for the MAE values given in Table 1, smaller values are obtained compared with the full dynamic range of the microarray spot (i.e., spot intensity values range is 1 to $2^{16}$).

To evaluate the performance of the proposed methodology for spot detection compared with the one already available, the means and the standard deviations of the distances between the centers of the Agilent annotations and the detected spots centers were computed for the entire datasets included in Table 1 and denoted by FEdata. Moreover, the accuracy of the detection denoted by the ratio of correctly identified microarray spots and the total number of spots was also computed. The results are included in Table 2, together with the results delivered by all approaches referenced in [52], employed for the detection of microarray spots disposed in both rectangular and hexagonal grids. A mean of 1.52 pixels with a standard deviation of less than 1 pixel and a spot detection accuracy of 100% underline the superior performance of our approach.

### 3.2. Shock Filters as a General Approach for Hexagonal-Grid-Layout Registration

Hexagonal grid layouts are becoming increasingly popular as the fields of nano- and meta-materials develop. In cell-cluster-array fabrication, self-assembled hexagonal superparamagnetic cone structures induce a local magnetic field gradient which inhibits the cancer cells’ migration [2]. For materials science applications, benefits such as increased optical performance and material resistance are added by hexagonal grid structures. Microlens arrays consisting of circular nanostepped pyramids disposed in hexagonal arrangements have showed efficient bidirectional light focusing and a maximal numerical aperture [4]. Considering the above cases, imaging techniques such as grid alignment and registration can be employed to determine the locations of image objects and to analyze and validate the respective structures of the materials in question.

**Figure 4:**
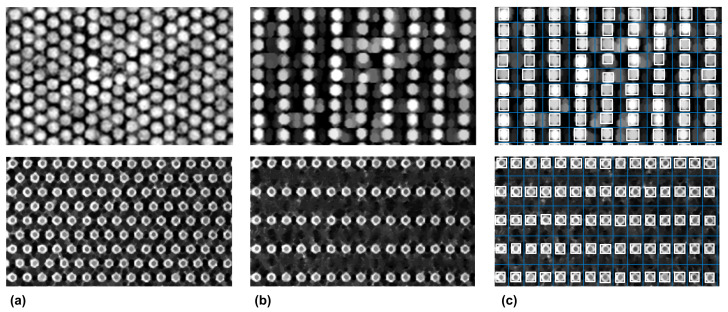
(**a**) Preprocessed images registered from hexagonal-array structure of pixelated CsI(Tl) scintillation screens (**top**) and hexagonal nanodisk–nanohole hybrid structure arrays (**bottom**). (**b**) Dual image decomposition based on shock filters driven by mathematical morphology; (**c**) segmentation.

Thus, in pixelated CsI(Tl) scintillation screens for X-ray imaging, the resolution for the pixelated screen with the hexagonal array structure is approximately 8.5% higher than for the screen with the square array structure [3]. Moreover, ultrathin hexagonal nanodisk–nanohole hybrid structure arrays have been employed for developing a novel plasmonic metasurface for subtractive color printing [62]. For the hexagonal-grid-layout image segmentation approaches, a crucial challenge is to develop a robust method which targets various types of hexagonal layout. In order to underline the generality of our proposed approach, both the hexagonal array structure of pixelated CsI(Tl) scintillation screens and the ultrathin hexagonal nanodisk-nanohole hybrid structure were processed using the proposed workflow. The obtained results are presented in Figure 4.

Regarding the main limitations of the proposed approach, the small size of the datasets considered for evaluation is mentioned. Nevertheless, the similarities between the segmentation accuracy metrics delivered by our approach and the ones delivered by the commercial Agilent Feature Extraction Software for over 100,000 microarray spots (Table 1) show the reproducibility of the results. The generality of the approach has also been proven by the results presented in Figure 4 for two other types of hexagonal grid layout. Since the propose approach was designed for microarray images, extensive testing and validation procedures are needed for segmentation procedures applied for other types of hexagonal-grid-layout images (e.g., ultrathin hexagonal nanodisk-nanohole hybrid structure arrays, pixelated CsI(Tl) scintillation screens), which are outside the the scope of current paper. Another drawback of the proposed method is that images with skewed, rotated or irregular hexagonal layouts require special attention. Rotation correction using the Radon transform is included within the proposed workflow, but irregular and skewed layouts are still not addressed. De-skewing algorithms are available [63,64], whereas for the irregular layouts, correction algorithms are to be designed based on the specifics of the irregularities.

### 3.3. Computational Complexity Analysis for the Hexagonal-Grid-Layout Image Segmentation

A large variety of image segmentation approaches are available, from complex ones such as deep learning approaches to reduced complexity ones which perform on image profiles, for example. Moreover, as detailed in [65,66], an interest in reducing the complexity of machine learning algorithms is shown. Thus, the user has to carefully evaluate the image analysis task and choose the appropriate processing approach. For hexagonal-grid-layout image segmentation, the computational complexity is estimated for the state-of-the-art approaches and compared with the proposed approach, in order to offer an overview of available methods from the computational complexity perspective. The comparison is detailed as follows.

Firstly, we estimated the computational complexity for our proposed approach for hexagonal-grid-layout microarray image registration. Considering a given M×N-pixel image, the obtained results are compared with both the classical state-of-the-art approaches [1,33,52] and the machine learning approaches [67,68] for microarray spot segmentation. The results are summarized in Table 3. In our case, the computational cost for the image registration procedure is detailed as follows:

(i) The morphological opening procedure and the autocorrelation spot size estimation cost are given by the upper bound function $f\left(M,N\right)=\left(2S_{e}MN+4MN\right)s$, with s representing one computational step, and $S_{e}$ representing the size of the structural element used for morphological filtering.
(ii) The computational complexity of the shock-filter-based procedure for grid alignment is based on the number of microarray spots found on each line and in each column of spots, denoted by α and β, respectively. Let *d* be the average of the microarray spot diameter and 2d be the average width for a line or a column of spots. We computed for each spot line and spot column, the horizontal and vertical image profiles, respectively, with the total complexity of 2αdM+2βdN=4MN. Shock filters were applied to each of the determined profiles having a complexity of p(αM+βN), where pαM represents *p* iterations performed on the number of α profiles (i.e., one profile for each line of spots), and each profile was of size *M*. This led to the estimation of the computational cost given by f(M,N)=6MNs+pd(αM+βN)s, with p>d. Consequently, the order of growth for the total computational cost was approximated to $O(2S_{e}MN+p\left(\alpha M+\beta N\right))$, and it represents the total computational complexity of the proposed method.

In [1,33] the Voronoi diagrams are used for the grid alignment in for hexagonal-grid-layout microarray images. According to the analysis performed in [69], the computational complexity is given by the order of growth of the computational cost $O\left(f\left(S\right)\right)=O\left(S^{2}log\left(S\right)\right)$, where *S* represents the total number of spots (i.e., for our images *S* = 44,000). The main disadvantage is that a unique region is obtained if weekly expressed spots are grouped together in the same area. This is overcome by the approach proposed in [52], where a preliminary step is added to the Voronoi diagram algorithm. This step detects all the highly expressed spots, which represent starting points for growing similar hexagonal areas for weakly expressed spots. In terms of the computational cost, the following term dMN is added to the cost function, leading to the total computational cost of $f\left(M,N,S\right)=S^{2}log\left(S\right)+dMN$.

Considering the machine learning approaches, the computational cost is given by both the training and prediction steps. Thus, according to Table 3, the order of growth for the machine learning-based grid alignment procedure has two terms, corresponding to the training and the prediction. For support vector machines, the total computational cost for grid alignment is given by $f\left(n,M,N\right)=n{\left(MN\right)}^{2}+nNM$, where *n* is the number of grid lines [70].

**Table 3:** Computational complexity analysis for microarray grid alignment.

Reference	Method	Cost Arguments	Order of Growth
[1,33]	Voronoi diagrams	*S*	$O(S^{2}\log S)$
[52]	Growing concentric hexagons	S,(M,N)	$O(S^{2}\log S+dMN)$
[43,71]	Support vector machines	(M,N)	$O(n{\left(MN\right)}^{2}+nMN)$
[72]	Evolutionary algorithms	S,(M,N)	$O(S^{2}+dMN)$
[67,68]	Deep neural Networks	-	-
present	Shock filters driven by morphology	$S_{e},\left(M,N\right)$	$O(2S_{e}MN+p\left(\alpha M+\beta N\right))$

Notes: *S*—represents total number of spots within the image under analysis (in round number 44,000); the pair (*M*, *N*) = (1650, 4320) corresponds to the image size in pixels; *S_e_* = 144 represents the size of the structuring element; the parameters denoted by lowercase letters are at least one order of magnitude smaller then the lowest one, *S_e_*.

For the evolutionary algorithms, the gridding approach for microarrays [72] differs from the classical ones, since it does not involve any 1D projection of the image. The approach includes a measure of fitness for possible grids to achieve a robust grid alignment against high levels of image noise and a high percentage of weakly expressed spots. Considering the fitness function, the evolutionary algorithm locates the regular grid that best fits a set of spot center coordinates. According to [73], as referred to the algorithm’s performance in terms of time complexity, the order of growth can be reduced to $O(m^{2}$), with *m* being the total number of graph edges. By approximating *m* with S (m>S), the total number of spots, and considering the preliminary computational steps which consist of image dilation and an approximate spot spacing calculation [72], the order of growth for the computational complexity of $O(S^{2}+dMN)$ is obtained.

Deep neural networks applied for microarray image analysis are discussed next in terms of computational complexity. To our knowledge, state-of-the-art research does not include deep neural networks applied for microarray grid alignment. Nevertheless, deep learning is used for bio-medical image segmentation [66], and, more precisely, it is also applied for microarray spot classification [68]. Since such approaches do not serve as grid alignment tools, the computational complexity levels of the deep learning approaches used for microarray spot segmentation were computed but not added to the Table 3 summary of grid alignment approaches [67,68]. Calling *s* the number of training samples, *f* the number of features and $n_{l_{i}}$ the number of neurons in layer *i*, we have the approximation for the computational complexity given by $O(s^{3}+fn_{l_{1}}+n_{l_{1}}n_{l_{2}}+\ldots )$, considering both the training procedure and the prediction. Taking into account the increased complexity, there is a great interest in reducing deep learning complexity, as shown in [66]. Herein, it is demonstrated that the computational complexity of the convolutional neural networks can be reduced by a factor of eight while achieving accurate bio-medical image segmentation. Even so, the computational complexity of our approach, which delivers segmentation information, as the results underline, is at least one order of magnitude lower than that of the deep learning approaches.

Let us consider the size of the image under analysis given by the (M,N) pair, with M=1650 and N=4320. As referred to in Table 3, we underline the cost arguments *S*, $S_{e}$ and *n* having the values 44,000, 144 and 120, respectively. Consequently, as denoted in Table 3, reduced computational complexity is achieved by the proposed grid-alignment approach, despite the iterative nature, considering that shock filters are applied on 1D image profiles. Thus, if the training procedure is excluded, the computational complexity of the proposed approach is similar to that of the support vector machine [43,71], whereas compared with the other approach, the computational complexity is at least one order of magnitude lower. It is to be noticed that the grid alignment is accurately performed for weekly expressed spots, due to the autocorrelation refinement procedure, and the accuracy is comparable with the machine learning approaches for grid alignment.

## 4. Conclusions

In this paper, we presented a novel segmentation approach for estimation of gene expression levels based on shock filters, making it applicable to both hexagonal and rectangular grid layouts. For hexagonal grids, the original image is divided into two rectangular grid images, such that their overlap constitutes the initial image. The proposed method was validated using specific quality measures such as the coefficient of variation and mean absolute error, on a dataset which includes hexagonal-grid-layout microarray images. The spot segmentation results obtained were compared with the ones delivered by Agilent Feature Extraction platform. Correlation coefficients between spot features (e.g., foreground intensity) and the mean distance between spot location showed very good agreement. Moreover, the computational cost of the described method was analyzed and compared with state-of-the-art microarray spot segmentation methods, ranging from classical to deep learning ones. Significantly lower computational complexity was achieved compared with the discussed methods. The segmentation accuracy, however, was comparable with those of machine learning approaches.

## Figures and Tables

**Table 1:** Evaluation of the image registration and segmentation accuracy.

Exp. ID	$r(I_{i},I_{i}^{\mathrm{FE}})$	${\mathrm{avg}}_{\mathrm{diff}}$	avg.MAE	$\mathrm{avg}.\;{\mathrm{MAE}}^{\mathrm{FE}}$	CV	${\mathrm{CV}}^{\mathrm{FE}}$
FE18398	0.988	0.075			0.420	0.412
FE18399	0.993	0.029			0.395	0.406
FE18400	0.982	0.093	536	524	0.414	0.385
FE18401	0.994	0.046			0.392	0.397

**Table 2:** Results of the proposed grid alignment methodology: means and standard deviations of distances between annotated and detected centers, and accuracy.

Reference/	Method Description	Image, Grid Type	Image Size (M,N)/	Spot	Metric	Value
Dataset			Number of Spots	Diam.		
SMD	Gridding based on support vector	Real, Rectangular grid	1980 × 1917	10	Mean	2.52
[42,60]	machines and genetic algorithms		9196		Std	2.59
					Acc	96.4
Nycter	K-nearest neighbor	Synthetic,	3188 × 9552	14	Mean	1.77
[61]		Rectangular grid	576,756		Std	1.16
					Acc	98.9
CNV370	Voronoi diagrams	Real, Rectangular grid	2800 × 2800	6	Mean	1.88
[52]			9216		Std	0.82
					Acc	99.8
Nycter	Gridding based on support vector	Real, Rectangular grid	2800 × 2800	14	Mean	1.91
	machines and genetic algorithms		9216		Std	1.03
					Acc	99.3
SMD	Voronoi diagrams	Real, Synthetic with	various sizes	14	Mean	1.94
Nycter		rectangular and			Std	2.32
[52]		hexagonal grids			Acc	97.5
FEdata	Shock filter driven by mathematical	Real, Hexagonal	1650 × 4320	14	Mean	1.52
(present)	morphology		9196		Std	0.68
					Acc	100
